# A novel chemical-combination screen in zebrafish identifies epigenetic small molecule candidates for the treatment of Duchenne muscular dystrophy

**DOI:** 10.1186/s13395-020-00251-4

**Published:** 2020-10-15

**Authors:** Gist H. Farr, Melanie Morris, Arianna Gomez, Thao Pham, Elisabeth Kilroy, Elizabeth U. Parker, Shery Said, Clarissa Henry, Lisa Maves

**Affiliations:** 1 Center for Developmental Biology and Regenerative Medicine, Seattle Children’s Research Institute, Seattle, WA USA; 2 Medical Student Research Training Program, University of Washington School of Medicine, Seattle, WA USA; 3 Molecular Medicine and Mechanisms of Disease Program, Department of Pathology, University of Washington, Seattle, WA USA; 4 Department of Pediatrics, University of Washington, Seattle, WA USA; 5 Graduate School of Biomedical Science and Engineering, University of Maine, Orono, ME USA; 6 School of Biology and Ecology, University of Maine, Orono, ME USA

**Keywords:** Chemical screen, Duchenne muscular dystrophy, Epigenetic small molecules, HDAC inhibitors, Zebrafish

## Abstract

**Background:**

Duchenne muscular dystrophy (DMD) is a severe neuromuscular disorder and is one of the most common muscular dystrophies. There are currently few effective therapies to treat the disease, although many small-molecule approaches are being pursued. Certain histone deacetylase inhibitors (HDACi) have been shown to ameliorate DMD phenotypes in mouse and zebrafish animal models. The HDACi givinostat has shown promise for DMD in clinical trials. However, beyond a small group of HDACi, other classes of epigenetic small molecules have not been broadly and systematically studied for their benefits for DMD.

**Methods:**

We used an established animal model for DMD, the zebrafish *dmd* mutant strain *sapje*. A commercially available library of epigenetic small molecules was used to treat embryonic-larval stages of *dmd* mutant zebrafish. We used a quantitative muscle birefringence assay in order to assess and compare the effects of small-molecule treatments on *dmd* mutant zebrafish skeletal muscle structure.

**Results:**

We performed a novel chemical-combination screen of a library of epigenetic compounds using the zebrafish *dmd* model. We identified candidate pools of epigenetic compounds that improve skeletal muscle structure in *dmd* mutant zebrafish. We then identified a specific combination of two HDACi compounds, oxamflatin and salermide, that ameliorated *dmd* mutant zebrafish skeletal muscle degeneration. We validated the effects of oxamflatin and salermide on *dmd* mutant zebrafish in an independent laboratory. Furthermore, we showed that the combination of oxamflatin and salermide caused increased levels of histone H4 acetylation in zebrafish larvae.

**Conclusions:**

Our results provide novel, effective methods for performing a combination of small-molecule screen in zebrafish. Our results also add to the growing evidence that epigenetic small molecules may be promising candidates for treating DMD.

## Background

Duchenne muscular dystrophy (DMD) is a severe neuromuscular disorder caused by mutations in the X-linked *DMD* gene, which encodes the protein dystrophin [1, 2]. Dystrophin is a key component of the dystrophin-associated protein complex (DAPC), a multi-protein assembly localized at the sarcolemma of muscle cells [3, 4]. Dystrophin and the DAPC serve a structural role in muscle cells, functioning as a stabilizing link between the cytoskeleton and extracellular matrix during muscle fiber contraction [5–7]. Dystrophin and the DAPC also regulate signaling pathways such as nitric oxide production and Ca^2+^ entry [6–9]. Loss of dystrophin and DAPC function makes muscle cell membranes susceptible to contraction-induced damage and leads to progressive calcium dysregulation, satellite cell dysfunction, inflammation, fibrosis, and necrosis [7, 10–13].

DMD is the most common type of muscular dystrophy, affecting approximately 1 in 3500-5000 male births [14, 15]. It first presents as motor difficulties in early childhood and progresses rapidly, leaving most affected boys in need of a wheelchair in their early teens and in need of respiratory aid in their 20s [16]. The disease is usually fatal in the third or fourth decade due to respiratory or cardiovascular failure.

Treatment options for DMD are still quite limited. The current standard of care is corticosteroid treatment, which delays the progression of muscle dysfunction but has serious side effects [16–18]. Besides corticosteroids, two other drugs are approved for a subset of DMD patients: eteplirsen, an exon-skipping oligonucleotide drug for patients with *DMD* exon 51 mutations, has received accelerated FDA approval in the USA, and ataluren, a stop codon read-through drug, has conditional approval in Europe for patients with *DMD* nonsense mutations [16, 19]. DMD gene therapy and gene editing approaches are very promising but face many challenges [20–23]. Many small-molecule approaches are being identified that could benefit DMD by modulating different pathological mechanisms downstream of the dystrophin mutation [7, 16, 19, 24]. A current view in the DMD field is that a combination of therapies targeting different pathological mechanisms may ultimately be most beneficial for patients [19, 24].

Histone deacetylase inhibitors (HDACi) are one class of small molecules that have shown promise in mouse and zebrafish DMD animal models and in DMD clinical trials (ClinicalTrials.gov NCT02851797, NCT03373968) [25–28]. HDACi are examples of “epigenetic small molecules,” compounds that target chromatin modifications and transcriptional regulators. Histone acetylation, which is often linked with open chromatin and active transcription, is one example of an epigenetic modification and is the target of many HDACi [29]. There are four different classes of histone deacetylase (HDAC) proteins, with different functions and tissue expression patterns, and different HDACi often selectively inhibit specific HDACs [30]. Studies in the *mdx* mouse model of DMD have identified epigenetic mechanisms involved in the pathogenesis of DMD, including constitutive activation of HDAC2 and dysregulation of certain histone modifications, leading to downstream transcriptional perturbations [31–34]. Dystrophin and the DAPC can regulate chromatin signaling through nitric oxide inhibition of HDAC2 [25, 32]. HDACi that have been shown to ameliorate pathology in the *mdx* mouse are the pan-HDACi Trichostatin A (TSA), valproic acid, phenylbutyrate, SAHA, and givinostat (ITF 2357), and the class I HDACi MS-275 [25, 28, 35–37]. TSA also ameliorates the zebrafish *dmd* mutant [27, 38], and givinostat has shown promise in DMD clinical trials [26]. However, beyond these HDACi, other classes of epigenetic small molecules have not been broadly and systematically tested in DMD models for their potential benefits for DMD.

Zebrafish are an outstanding animal model for DMD [39–43]. A zebrafish *dmd* mutant strain, *dmd*^*ta222a*^, also known as *sapje*, has a nonsense mutation in exon 4 and is a dystrophin-deficient model of DMD [39, 41, 44]. The zebrafish *dmd*^*ta222a*^ mutation is autosomal recessive, and, following a Mendelian ratio, approximately 25% of the offspring from a heterozygous *dmd*+/− cross exhibit degenerative muscle lesions by about 3 days post-fertilization. *dmd* mutant zebrafish exhibit many aspects of human DMD pathology; in particular, skeletal muscle fibrosis and inflammation, including infiltration of mononuclear cells [40, 41].

*dmd* mutant zebrafish offer several advantages for screening and evaluating small molecule therapies [42, 43]. Zebrafish eggs can be rapidly produced in large numbers, and the resulting embryos readily absorb small molecules. Because skeletal muscle lesions can be observed within 3-4 days of development, *dmd* mutant zebrafish are amenable to rapid and high-throughput screening. An exceptional range of approaches are available for assessing treatment outcomes in *dmd* mutant zebrafish, including assays of skeletal muscle structure and function as well as survival [42, 43, 45, 46]. In particular, muscle structure can easily be observed in zebrafish larvae using polarized light birefringence techniques [44, 45, 47]. Large-scale chemical screens have highlighted the potential of *dmd* mutant zebrafish for identifying new therapeutic compounds and targets as well as for understanding the molecular mechanisms behind DMD [45, 48, 49]. These large-scale screens, which applied either pools of eight chemicals or individual chemicals to *dmd* mutant zebrafish, have tested over 4000 compounds and identified 25 positive hits [43]. In addition, some small molecules, such as TSA, have been shown to ameliorate both *dmd* mutant zebrafish and *mdx* mice [27, 45, 50–52]. Because of this strong conservation, insight from zebrafish can inform our understanding of human DMD disease, while also taking advantage of the utility of high-throughput analysis.

Here, we performed a pilot screen of the commercially available Cayman Chemical Epigenetics Screening Library to identify additional epigenetic small molecules that could improve the *dmd* mutant zebrafish muscle phenotype. We identified candidate pools of epigenetic compounds that improve skeletal muscle structure in *dmd* mutant zebrafish. We then identified a specific combination of two HDACi compounds, oxamflatin and salermide, that ameliorated *dmd* mutant zebrafish skeletal muscle degeneration. We validated the effects of oxamflatin and salermide on *dmd* mutant zebrafish in an independent laboratory. Furthermore, we showed that the combination of oxamflatin and salermide caused increased levels of histone H4 acetylation in zebrafish larvae. Overall, our study provides novel and effective methods for performing a pooled chemical-combination screen and gives further evidence that epigenetic small molecules may be good candidates for successfully treating DMD.

## Methods

### Zebrafish husbandry

All experiments involving live zebrafish (*Danio rerio*) were carried out in compliance with Seattle Children’s Research Institute’s and the University of Maine’s Institutional Animal Care and Use Committee guidelines. Zebrafish were raised and staged as previously described [53]. Time (hpf or dpf) refers to hours or days post-fertilization at 28.5 °C. Eggs were collected from 20-30-min spawning periods and raised in Petri dishes in ICS water (300 mg Instant Ocean/L, 0.56 mM CaCl2, 1.2 mM NaHCO3), in a dark 28.5 °C incubator, up to 5 dpf. After 5 dpf, fish were maintained on a recirculating water system (Aquaneering) under a 14-h on, 10-h off light cycle. From 6-30 dpf, fish were raised in 2.8 L tanks with a density of no more than 50 fish per tank and fed a standard diet of paramecia (Carolina) one time per day and Zeigler AP100 dry larval diet two times per day. From 30 dpf on, fish were raised in 6 L tanks with a density of no more than 50 fish per tank and fed a standard diet of *Artemia nauplii* (Brine Shrimp Direct) and Zeigler adult zebrafish feed, each two times per day. The wild-type stock and genetic background used was AB. The zebrafish *dmd*^*ta222a*^ mutant line (also known as *sapje*; hereafter, referred to as *dmd*) has been described and is a null allele [39, 44]. *dmd*^*ta222a*^ genotyping was performed as previously described [54].

### Small molecules

**Fig. 1:**
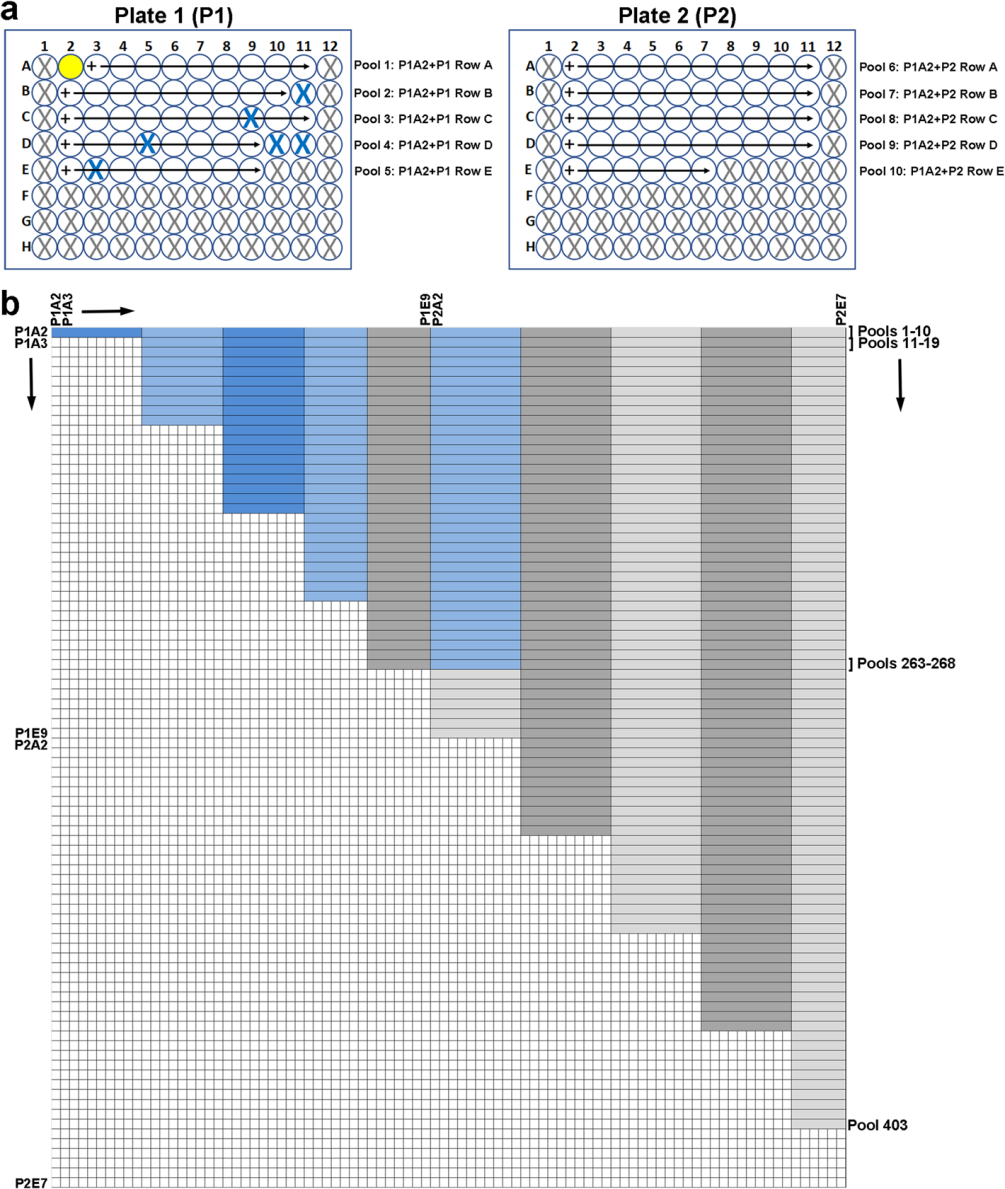
Cayman Chemical Epigenetic Screening Library chemical-combination pooling scheme. **a** The library consists of 2 96-well plates (P1, P2). See Table 1 for the list of chemicals. Gray X’s mark empty wells. Blue X’s mark wells with chemicals not used in our screen: CAY10669, (−)-JQ1, MI-nc, GSK-J1, GSK-J2, and GSK-J5. Pooling process for plate 1 well A2 chemical (3-amino benzamide; yellow well) is illustrated. **b** Grid representation of the library chemicals and pools, in which each chemical (88 out of 94 total library chemicals) is represented along both the *x*- and *y*-axes. CAY10669, (−)-JQ1, MI-nc, GSK-J1, GSK-J2, and GSK-J5 are removed from this grid representation. Shading (dark blue/light blue, dark gray/light gray) represents rows of chemicals in the library plates. Each horizontal, alternately shaded row represents a candidate chemical pool. Blue shading represents the 93 pools tested. Gray shading represents the remaining candidate pools (403 total pools on this grid)

Epigenetic small molecule library screening was performed using the Cayman Chemical Epigenetics Screening Library (Item No. 11076, Batch No. 0455098). The library was received as 10 mM stocks of each chemical dissolved in dimethyl sulfoxide (DMSO). The composition of the Cayman Chemical Epigenetics Screening Library can vary. The version we obtained contained 94 chemicals distributed over two 96-well plates. The identities of the chemicals and plate layouts are shown in Table 1 and Fig. 1a. Additional chemicals were purchased individually from Cayman Chemical and were dissolved in DMSO (Sigma).

**Table 1:** Cayman chemical epigenetics screening library

Plate 1		Plate 2	
Well	Small molecule	Well	Small molecule
A2	3-amino benzamide	A2	AGK2
A3	SB 939	A3	CAY10603
A4	PCI 34051	A4	Chaetocin
A5	4-iodo-SAHA	A5	Splitomicin
A6	Sirtinol	A6	CBHA
A7	C646	A7	M 344
A8	Ellagic acid	A8	Oxamflatin
A9	Scriptaid	A9	Salermide
A10	Suberohydroxamic acid	A10	Mirin
A11	Apicidin	A11	Pimelic diphenylamide 106
B2	UNC0321 (trifluoroacetate salt)	B2	(S)-HDAC-42
B3	(−)-Neplanocin A	B3	MS-275
B4	Cl-Amidine	B4	HNHA
B5	F-Amidine (trifluoroacetate salt)	B5	RG-108
B6	JGB1741	B6	2′,3′,5′-triacetyl-5-azacytidine
B7	UNC0638	B7	S-Adenosylhomocysteine
B8	Phthalazinone pyrazole	B8	UNC0224
B9	Isoliquiritigenin	B9	Chidamide
B10	CCG-100602	B10	3-Deazaneplanocin A
B11	CAY10669	B11	Sinefungin
C2	Zebularine	C2	Pyroxamide
C3	Delphinidin chloride	C3	WDR5-0103
C4	ITF 2357	C4	AMI-1 (sodium salt)
C5	PFI-1	C5	UNC1215
C6	5-Azacytidine	C6	GSK 343
C7	Decitabine	C7	SIRT1/2 inhibitor IV
C8	(+)-JQ1	C8	I-CBP112 (hydrochloride)
C9	(−)-JQ1	C9	UNC1999
C10	BSI-201	C10	PFI-3
C11	1-Naphthoic acid	C11	*trans*-Resveratrol
D2	Sodium 4-phenylbutyrate	D2	2,4-DPD
D3	IOX1	D3	DMOG
D4	MI-2 (hydrochloride)	D4	Trichostatin A
D5	MI-nc (hydrochloride)	D5	CAY10398
D6	Gemcitabine	D6	RSC-133
D7	Lomeguatrib	D7	KD 5170
D8	Octyl-α-ketoglutarate	D8	CAY10433
D9	Daminozide	D9	Piceatannol
D10	GSK-J1 (sodium salt)	D10	CAY10591
D11	GSK-J2 (sodium salt)	D11	EX-527
E2	GSK-J4 (hydrochloride)	E2	SAHA
E3	GSK-J5 (hydrochloride)	E3	2-PCPA (hydrochloride)
E4	CPTH2	E4	Nicotinamide
E5	Valproic acid (sodium salt)	E5	BIX01294
E6	Tenovin-1	E6	N-Oxalylglycine
E7	Tenovin-6	E7	Suramin (sodium salt)
E8	Sodium butyrate		
E9	Anacardic acid		

The identities of the chemicals and composition of the library, 94 chemicals distributed over two 96-well plates, are shown. The library (Cayman Chemical Item No. 11076, Batch No. 0455098) was received as 10 mM stocks of each chemical in DMSO

### Small molecule treatments

To test toxicity and assess working doses of the Epigenetics Screening Library chemicals, wild-type AB embryos were treated with individual chemicals beginning at 4 hpf. Embryos were not dechorionated prior to these dose tests. Three concentrations of each chemical were tested: 10 μM, 1 μM, and 100 nM. Embryos were treated with small molecules with 1% DMSO in embryo medium (EM; 14.97 mM NaCl, 0.50 mM KCl, 0.98 mM CaCl_2_.2H2O, 0.15 mM KH_2_PO_4_, 0.99 mM MgSO_4_.7H2O, 0.05 mM Na_2_HPO_4_, 0.83 mM NaHCO_3_), or with 1% DMSO in EM as a vehicle control. One chemical from the library, JGB1741, exhibited precipitation from EM at 10 μM but not at lower concentrations; all other chemicals appeared soluble with DMSO in EM. Twelve-well plates were used for treatments; each well contained 25 embryos and 3 mL of chemical treatment or vehicle control. The treatment media was changed every 24 h until 4 dpf. Over the 4-day treatments, developmental abnormalities and survival were noted. Most chemical treatments showed little or no effect on embryo health and survival at 1 μM doses, so we determined 1 μM to be the working dose for pooled screening for most of the library chemicals. C646 and apicidin were toxic and caused embryo lethality at 1 μM but not at 100 nM, so these compounds were used at 100 nM. CAY10669 exhibited strong toxicity and GSK-J1 is reported to be non-cell permeable, so these chemicals were removed from chemical pool screening. (−)-JQ1, MI-nc, GSK-J2, and GSK-J5 are designated as negative control compounds in the library and thus were also removed from chemical pool screening.

For chemical screening and *dmd* rescue tests, embryos from *dmd+/−* crosses were used. At about 24 hpf, the chorions were manually removed from the embryos, and embryos were sorted into wells of 12-well plates for treatments. Each well contained 25 embryos and 3 mL of chemical treatment or vehicle control. 1000X stocks of chemicals were made in DMSO just prior to each treatment experiment. Embryos were treated with chemicals with 1% DMSO in embryo medium (EM), or with 1% DMSO in EM as a vehicle control. Treatments were started at about 24 hpf and continued for 3 days, changing the treatment or vehicle control EM solution every 24 h. Larvae were fixed in 4% paraformaldehyde (PFA) in PBS at 4 dpf and stored at 4 °C. To obtain tissue for genotyping, larval heads were removed with a scalpel at the level of the pectoral fins. Larval tails with trunk skeletal muscle remained intact and were maintained in 4% PFA until imaging was performed.

### Imaging and scoring muscle lesions

Larvae fixed in 4% PFA were rinsed in PBS with 0.01% Tween (PBSTw) prior to imaging. Animals were placed in PBSTw in a 60-mm glass Petri dish. An Olympus SZX16 stereomicroscope with an attached Olympus DP72 camera was set up with one sheet of polarizing film over the trans-illumination base and another sheet of the film placed over the objective lens such that the two films were crossed [55]. For chemical pool screening, larvae were sorted under birefringence into muscle lesion positive or negative groups. The number of larvae in each group was recorded.

For quantitative birefringence measurements, we adapted an approach based on previously described methods [47, 55]. In this approach, larvae were placed in a glass-bottom Petri dish in 2.5% methyl cellulose and oriented to maximize the brightness of the muscle tissue through the crossed polarizers. If necessary, pectoral fins and any remaining yolk tissue were removed so that the larvae could lie flat, to ensure consistent birefringence across the length of the trunk. Birefringence was imaged in individual animals as above. Images were acquired using the Olympus Cellsens Dimensions software. Exposure time was adjusted so that only a few saturated pixels were present. Adjusting exposure time in this way should control for any variation in brightness caused by larvae being at slightly different orientations. All other microscope and camera settings were kept constant. ImageJ was used to outline the trunk musculature, using the wand tool, and to calculate the average pixel intensity within the resulting selection. Average pixel intensity values for experimental conditions were normalized to wild-type control values. We see similar relative average pixel intensity values for control and mutant samples from experiment to experiment, indicating that our procedure is highly reproducible.

### Wholemount immunocytochemistry and phalloidin staining

Whole-animal immunostaining was performed with the primary antibodies anti-dystrophin (1:100, Sigma D8043) and anti-beta dystroglycan (1:50, Novocastra NCL-b-DG). The secondary antibody was either goat anti-mouse AlexaFluor-488 or goat anti-mouse AlexaFluor-568 (1:200, Life Technologies/Molecular Probes). Four dpf larvae were fixed in 4% PFA in PBS for 4 h at room temperature and then washed into PBSTw. For anti-dystrophin staining, larvae were dehydrated through a methanol series and stored in 100% methanol at −20 °C overnight. They were then re-hydrated into PBSTw, washed 5× in PBSTw containing 1% DMSO, and blocked overnight at 4 °C in 2% BSA, 2% heat inactivated normal goat serum, and 1% DMSO in PBSTw. The samples were then incubated with primary antibody diluted in the above block for 24 h at 4 °C, washed with PBSTw +1% DMSO, re-blocked for 6 h at room temperature, incubated with secondary antibody for 16 h at 4 °C, washed with PBSTw +1% DMSO, and stored in 4% PFA in PBS. For anti-beta dystroglycan staining, the fixed larvae were permeabilized with 2% Triton X-100 in PBS for 2 h at room temperature, then washed into PBSTw, and then blocked in 5% BSA, 2% heat-inactivated normal goat serum, 1% DMSO, 1% Triton X-100, and 0.2% saponin in PBS overnight at 4 °C. The samples were then processed as for the anti-dystrophin stained samples, except the block including Triton X-100 and saponin was used. To visualize actin in the muscle fibers of the beta-dystroglycan-stained animals, CF488A-conjugated phalloidin (1:50, Biotium Cat. # 00042) was included with the secondary antibody. For confocal imaging, the heads and yolk were removed from representative larvae and the trunks/tails were mounted laterally in 80% glycerol containing 4% propyl gallate. Images were collected using a Leica TCS SP5 II confocal on a DMI6000 stand with a 20× NA 0.7 air objective.

### Immunoblotting

Chemical treatments of embryos from *dmd+/−* crosses were performed as above. At 4 dpf, heads were cut from anesthetized animals and were arrayed in 96-well plates for genotyping. The remaining trunk and tail portions of the animals were arrayed in separate 96-well plates, to which LDS sample buffer (Invitrogen NP0007) was added and stored at −20^0^C until genotyping was completed. Trunk lysates were then pooled by genotype. Approximately, 0.75 animal equivalent per lane was separated on 12% NuPAGE Bis-Tris gels run in NuPAGE MES buffer (Invitrogen) and blotted. Blots were probed separately with anti-pan acetylated histone H4 (1:2000, rabbit polyclonal, Millipore Cat. # 06-866) and anti-acetylated histone H4 K16 (1:2500, rabbit polyclonal, Millipore Cat. # 07-329). Anti-actin was included with both anti-acetylated histone antibodies as a loading control (1:2000; mouse monoclonal Clone C4, MP Biomedical Cat. # 69100). Infrared-dye labeled secondary antibodies (Rockland) were visualized using a Licor Odyssey infrared scanner.

### Statistical analyses

Statistical tests used are provided in the figure legends. Statistical analyses were performed and graphs were constructed using GraphPad Prism 8.

## Results

### Epigenetic small-molecule library pooling approach

In a previous study, we showed that the pan-HDACi TSA could improve the zebrafish *dmd* muscle lesion phenotype [27]. We wanted to test whether we could identify new epigenetic small molecules, or epigenetic small molecule combinations, that improve the zebrafish *dmd* muscle lesion phenotype. We thus decided to test the effects of small molecules from the commercially available Cayman Chemical Epigenetics Screening Library. The version of the library that we obtained contained 94 chemicals distributed over two 96-well plates (Table 1 and Fig. 1a). Prior to screening these chemicals on *dmd* mutant embryos, we performed dose and toxicity testing of each individual library compound on wild-type embryos (see the “Methods” section). Most chemicals showed little or no detrimental effect on embryo health and survival at a 1 μM dose, so we selected 1 μM to be the working dose for chemical screening for most of the library compounds. C646 and apicidin were toxic and caused embryo lethality at 1 μM but not at 100 nM, so these compounds were used at 100 nM. CAY10669 exhibited strong toxicity and embryonic lethality by 3 dpf (days post-fertilization) and was removed from further screening. GSK-J1 is reported to be non-cell permeable and was removed from further screening (Fig. 1a). We also removed library chemicals that are designated as negative control compounds from further screening ((−)-JQ1, MI-nc, GSK-J2, and GSK-J5; Table 1; Fig. 1a).

Previous large-scale chemical screens in *dmd* mutant zebrafish either tested individual chemicals or tested chemical pools by subdividing libraries into unique chemical pools containing eight compounds each [45, 48, 49]. In contrast to these previous screens, we wanted a strategy that would test each compound in combination with every other library compound. In order to efficiently test combinations of epigenetic small molecules in the library, we designed a grid system consisting of a set of 403 different chemical pools (Fig. 1a, b). In this system, compound A2 of plate 1 is pooled with the remaining compounds of row A of plate 1 for combination pool 1 (Fig. 1a, b). Compound A2 is then pooled with compounds from plate 1 row B (pool 2), plate 1 row C (pool 3), and the remaining rows from the library for pools 4-10 (Fig. 1a, b). Compound A3 of plate 1 is then combined with compounds of plate 1 row B for pool 11, and so on (Fig. 1a, b). In this grid system, each compound would be tested in combination with each of the other library chemicals in at least 1 pool (Fig. 1a, b).

**Fig. 2:**
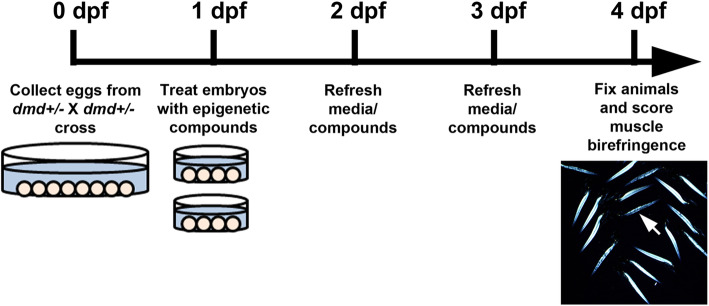
Timeline of *dmd* mutant zebrafish small molecule treatments. Eggs are collected from crosses of *dmd+/−* fish. *dmd* is autosomal recessive in zebrafish, so about 25% of embryos will be *dmd−/−*. At 1 dpf (days post-fertilization), embryos are sorted into dishes for treatments, and DMSO or small molecule compounds are added to the embryo bath (as in [27]). Treatment media are replaced daily. At 4 dpf, animals are fixed and muscle birefringence is scored. *dmd−/−* animals exhibit dark lesions in the larval trunk muscle (arrow in image), as visualized using polarized light birefringence

**Fig. 3:**
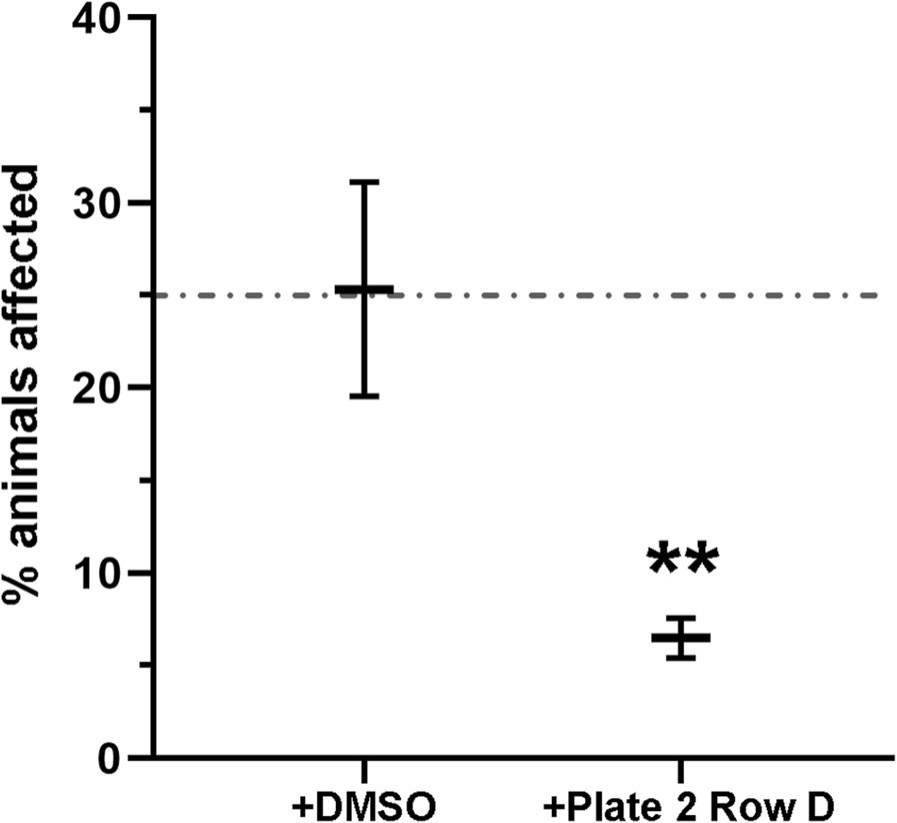
Plate 2 row D chemical pool, which includes TSA, significantly reduces the frequency of zebrafish *dmd* mutant animals exhibiting abnormal muscle birefringence. Control treatment is 1% DMSO. The dashed line represents the expected 25% affected animals. See Table 1 for the 10 chemicals in plate 2 row D. Each chemical was used at 1 μM. For each treatment condition, *n* = 3 replicates, with 23-27 embryos in each replicate. Error bars represent standard error. Significance was determined using a Mantel-Haenszel test. **p < 0.004 compared to DMSO control

To determine if screening pools of epigenetic small molecules was sensitive enough to identify compounds that could improve the zebrafish *dmd* muscle phenotype, we tested the pool of 10 chemicals from plate 2 row D, which contains TSA (Table 1). Animals from *dmd+/−* crosses were treated from 1-4 days and then scored for muscle birefringence (as in [27] and illustrated in Fig. 2). Treatment with the pool of chemicals from plate 2 row D significantly decreased the number of affected animals exhibiting abnormal muscle birefringence (Fig. 3). These results indicate that a pool of compounds containing a known beneficial epigenetic small molecule, TSA, can improve the zebrafish *dmd* muscle phenotype. These results suggest that the epigenetic chemical-pooling design of our screen is sensitive enough to pick up positive hits of small molecules that improve the zebrafish *dmd* muscle phenotype.

### Pilot screen of epigenetic small-molecule combination pools

**Fig. 4:**
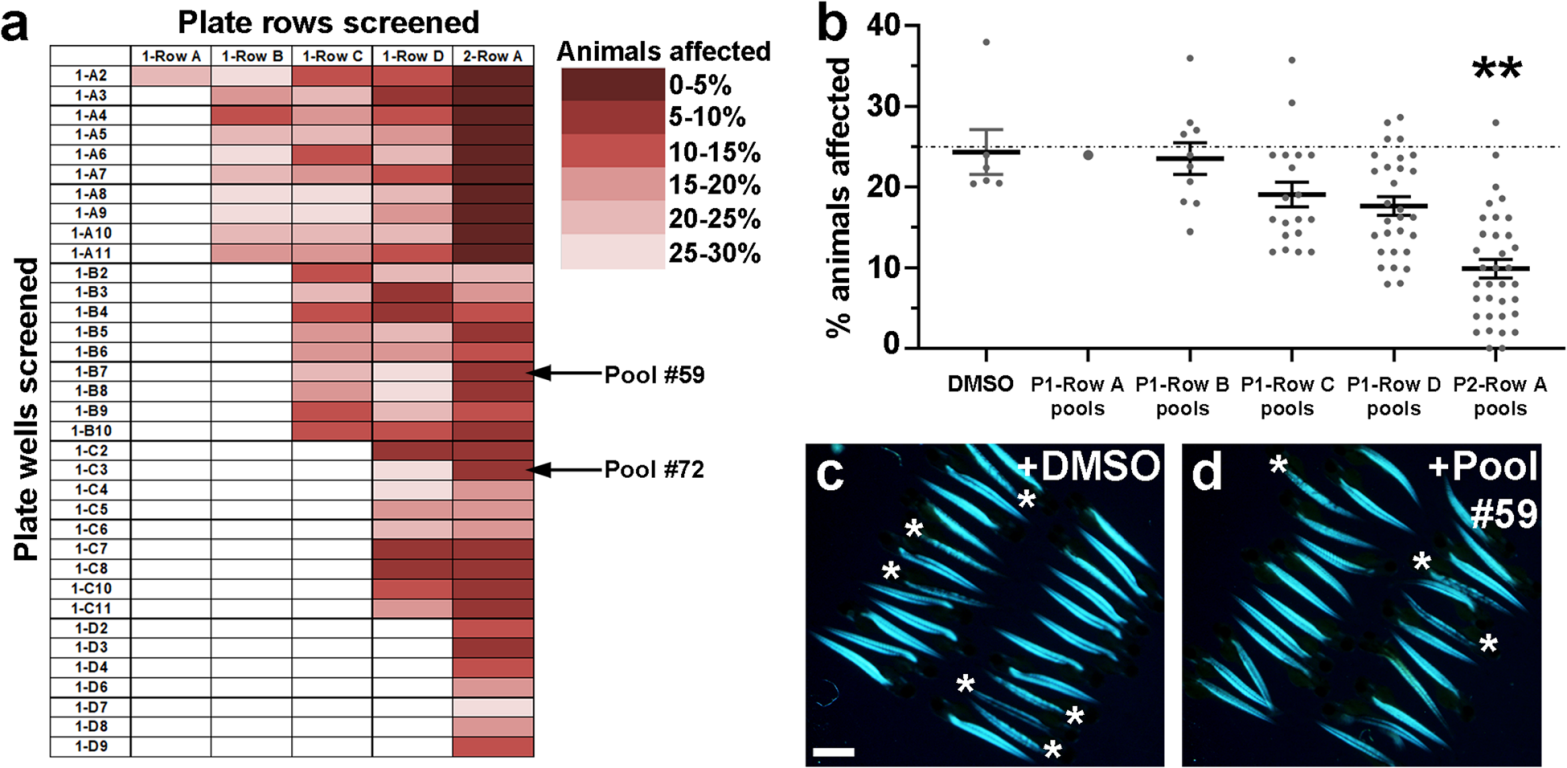
Pilot screen identifies the ability of plate 2 row A chemical pools to lower the frequency of affected *dmd* animals. (**a**) Heat map representation of the average percentage of affected animals observed from each of the 93 pools tested. Individual chemical wells tested were from plate 1 (left column, plate number and well number shown). Library rows tested are rows A-D from plate 1 and row A from plate 2. See Table 1 for the chemicals in each well and row. Arrows point to pilot screen pool #59 (pool 135 in Fig. 1b), shown in (**d**), and pilot screen pool #72 (pool 173 in Fig. 1b), analyzed in Fig. 5 below. (**b**) Graph of combined average percentages of affected animals from all tested pools that included each plate row. Control treatments are 1% DMSO. The dashed line represents the average of the control DMSO treatments (25%). Each dot represents a tested drug pool’s average of affected animals. Error bars represent standard error. Significance was determined using a one-way ANOVA test comparing each chemical pool group to the DMSO control group with Dunnett’s correction for multiple comparisons. **p = 0.0021 compared to DMSO control. (**c**, **d**) Example of birefringence images of 4 dpf larvae from a replicate of (**c**) DMSO control and (**d**) pool #59 (plate 2 row A + UNC0638) treatments. DMSO control animals are siblings of pool #59 animals. Asterisks mark affected animals. Scale bar = 1 mm

We then proceeded to test epigenetic small molecule pools from our chemical-combination grid system (Fig. 1). We tested 93 of the 403 possible chemical pool combinations (Figs. 1b and 4a). Animals from *dmd+/−* crosses were treated from 1-4 days and then scored for muscle birefringence. Our approach for assessing the dystrophic phenotype using birefringence in our pilot library screen followed the approach used in previous zebrafish *dmd* mutant chemical screens [45, 48, 49], in which control and drug-treated animals are scored as “affected,” or showing a clear *dmd* mutant muscle lesion phenotype by birefringence, or “unaffected,” or showing largely normal birefringence. Each pool was tested in duplicate, with 25 animals per well for each replicate. We determined the average percentage of affected animals from each chemical pool, and from each DMSO control tested, and we plotted the results as a heat map on our combination pool grid (Fig. 4a). This heat map grid reveals that the majority of pools tested that included plate 2 row A reduce the percentage of animals affected (column 5 in Fig. 4a). To test whether the plate 2 row A pools are significantly improving the percentage of animals affected, we plotted the combined results from all pools containing each plate row compared with the combined results from DMSO control treatments (Fig. 4b). This analysis shows that treatment pools using plate 2 row A led to a significant reduction in the percentage of affected animals relative to the DMSO controls (Fig. 4b). On average, treatment with chemical pools that included plate 2 row A resulted in only about 10% affected animals, compared to the 25% affected average observed in the DMSO controls (Fig. 4b). Figure 4c and d shows an example of the improved birefringence from animals treated with a representative pool from the plate 2 row A pools (pool 135 in Fig. 1b; pilot screen pool #59), compared to sibling DMSO controls. While these results do not rule out beneficial effects of compounds or treatment pools from our screen that did not include plate 2 row A, these results do highlight that compounds within plate 2 row A are having significant beneficial effects on the zebrafish *dmd* muscle lesion phenotype.

### Analysis of plate 2 row A small molecules identifies a beneficial combination of oxamflatin and salermide

**Fig. 5:**
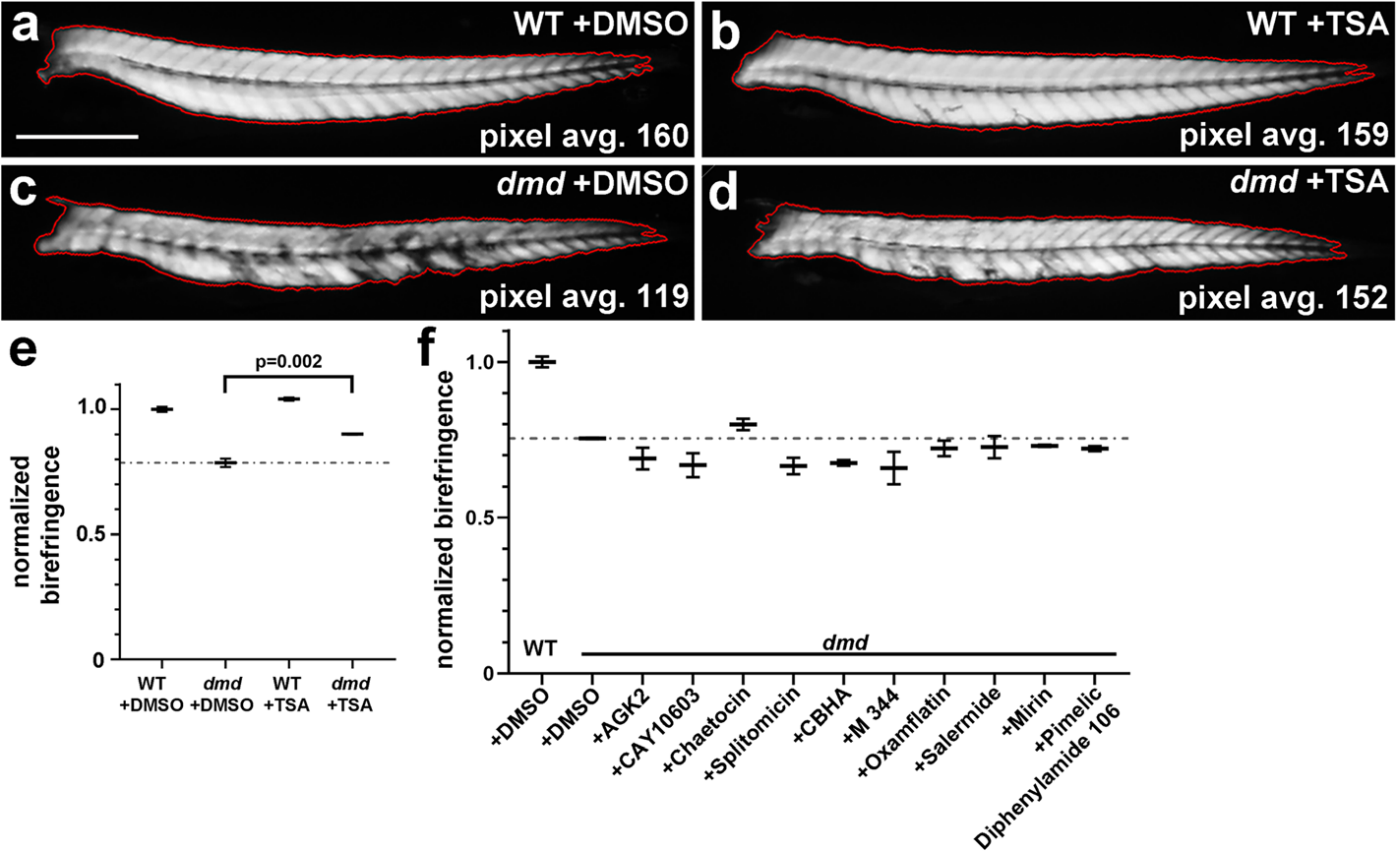
Individual compounds from plate 2 row A do not significantly improve zebrafish *dmd* mutant muscle birefringence. **a**-**d** Four dpf zebrafish trunk muscle, with trunk skeletal muscle birefringence outlined in red. Lateral views, anterior to the left. Average pixel brightness values are shown for (**a**) wild-type (WT) + DMSO, (**b**) WT + TSA, (**c**) *dmd* + DMSO, and (**d**) *dmd* + TSA. Scale bar = 500 μm. **e** Graph of normalized birefringence pixel intensities for 200 nM TSA treatments vs DMSO controls. *n* = 3 biological replicates for each treatment. Plot shows the average normalized pixel intensity for the 3 replicates for each treatment (4-9 genotyped animals per replicate). The dashed line represents the average normalized pixel intensity for all of the DMSO-treated *dmd* animals (*n* = 17). Error bars represent standard error. *p* value determined by Student’s *t* test. (**f**) Graph of normalized birefringence pixel intensities for treatments of zebrafish *dmd* mutants with the 10 individual chemicals from plate 2 row A. Control treatment is 1% DMSO. All chemicals were tested at 1 μM. For each treatment condition, *n* = 3 replicates, with 2-9 *dmd* embryos in each replicate. Plot shows the normalized birefringence pixel intensity for the 3 replicates for each treatment. The dashed line represents the average normalized pixel intensity for all of the DMSO-treated *dmd* animals (*n* = 15). Error bars represent standard error. Significance was determined using a one-way ANOVA test comparing each treatment group to the *dmd* DMSO control group with Dunnett’s correction for multiple comparisons. *p* > 0.2 for all individual chemicals compared to *dmd* DMSO control

While useful for screening purposes, the approach of scoring animals as “affected” versus “unaffected” may only provide an approximate measure of the effects of small molecules on the *dmd* mutant phenotype, as animals scored as “affected” could still be improved over typical *dmd* mutants, and “unaffected” larvae could still have dystrophic lesions but are substantially less severe than is typical for *dmd* mutants. To determine which compounds mediate the effect of plate 2 row A, we wanted an approach that would allow us to more quantitatively assess the effects of small molecules on zebrafish *dmd* mutant birefringence. Adapting previously described methods [47, 55], we used gray value measurements to quantitate the brightness level of the muscle birefringence of each animal (Fig. 5a). For each animal, we outlined a unilateral area of trunk muscle birefringence and measured the average pixel brightness within that area (Fig. 5a). Zebrafish *dmd* mutant animals show reduced birefringence, as measured by lower pixel intensity, compared to their control siblings (Fig. 5c, e). As a test of this approach, we confirmed that TSA treatment significantly improved the average pixel brightness of *dmd* mutant birefringence (Fig. 5d, e).

We, next tested each compound from plate 2 row A to see if any of the individual chemicals could improve the *dmd* birefringence phenotype. Animals from *dmd+/−* crosses were treated from 1-4 days and then scored for muscle birefringence. When tested individually, none of the compounds showed a significant effect on the DMSO-treated *dmd* muscle birefringence brightness (Fig. 5f). These results indicate that a combination of chemicals from plate 2 row A is needed to improve the *dmd* mutant birefringence phenotype.

**Fig. 6:**
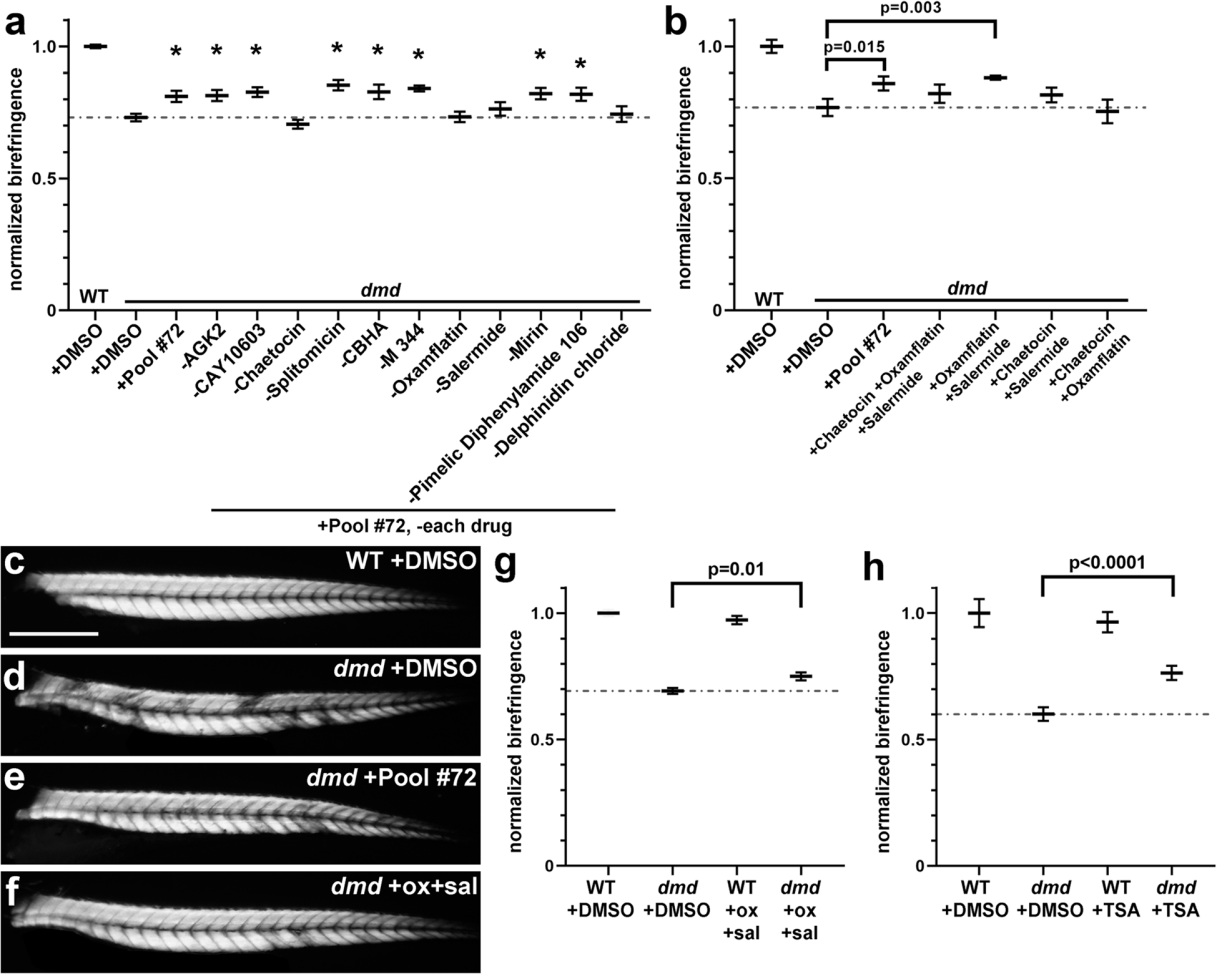
Oxamflatin and salermide mediate the effects of plate 2 row A. **a** Graph of average pixel intensities for treatments of zebrafish *dmd* mutants with pilot screen pool #72 and with pool #72 with each individual chemical removed. Control treatment is 1% DMSO. All chemicals included in the pools were tested at 1 μM. For each condition, 3 replicates of 25 embryos each were treated, with 2-9 *dmd−/−* embryos in each replicate. Plotted are the average normalized pixel intensities of the embryos from all 3 replicates for that treatment (*n* = 14-26 total *dmd−/−* embryos per treatment). Treatment with pool #72 without chaetocin, oxamflatin, salermide, or delphinidin chloride did not achieve the rescue effect seen with the full pool #72. The dashed line represents the average normalized pixel intensity for all of the DMSO-treated *dmd* animals (*n* = 26). Error bars represent standard error. Significance was determined using a one-way ANOVA test comparing each treatment group to the *dmd* DMSO control group with Dunnett’s correction for multiple comparisons. *p ≤ 0.04 compared to *dmd* DMSO control. **b** Graph of average normalized pixel intensities for treatments of *dmd* mutants with pool #72 and with combinations of chaetocin, oxamflatin, and salermide. Control treatment is 1% DMSO. All chemicals were used at 1 μM. For each treatment condition, *n* = 3 replicates, with 1-11 *dmd−/−* embryos in each replicate. Plot shows the average normalized pixel intensity for the 3 replicates for each treatment. The dashed line represents the average normalized pixel intensity for all of the DMSO-treated *dmd* animals (*n* = 14). Error bars represent standard error. Significance was determined using a one-way ANOVA test comparing each treatment group to the *dmd* DMSO control group with Dunnett’s correction for multiple comparisons. **c-f** 4 dpf zebrafish trunk muscle birefringence. Lateral views, anterior to the left. Representative animals are shown from treatments in (**b**). **c** WT + DMSO, **d** *dmd* + DMSO, **e** *dmd* + pool #72, and **f** *dmd* + oxamflatin and salermide. Scale bar = 500 μm. **g** Validation of oxamflatin and salermide treatment effects. Graph of average normalized pixel intensities for treatments of *dmd* fish with oxamflatin and salermide, performed in the Henry Lab. Treatments were performed from 1-4 dpf. Control treatment is 1% DMSO. Compounds were each used at 1 μM. Dashed line represents the average normalized pixel intensity for all of the DMSO-treated *dmd−/−* animals (*n* = 11). WT + DMSO, *n* = 10; WT + ox+sal, *n* = 9; *dmd−/−* + ox+sal, *n* = 14. Error bars represent standard error. *p* value determined by Student’s *t* test. **h** Validation of TSA treatment effects. Graph of average normalized pixel intensities for treatments of *dmd* fish with TSA, performed in the Henry Lab. Treatments were performed from 1-4 dpf. Control treatment is 1% DMSO. TSA was used at 200 nM. Dashed line represents the average normalized pixel intensity for all of the DMSO-treated *dmd−/−* animals (*n* = 31). WT + DMSO, *n* = 6; WT + TSA, *n* = 6; *dmd−/−* + TSA, *n* = 28. Error bars represent standard error. *p* value determined by Student’s *t* test

To identify the compounds required for the effects of plate 2 row A, we next decided to remove individual chemicals from the drug pool. We chose to test pilot screen pool #72 (pool 173 in Fig. 1b; plate 2 row A + plate 1 well C3; 11 total chemicals), because this pool represented an average effect from the plate 2 row A pools (Fig. 4a, b). As above, animals from *dmd+/−* crosses were treated from 1-4 days and then scored for muscle birefringence. Treatment with the full 11 chemicals from pool #72 showed significant improvement of the *dmd* mutant birefringence using our quantitative analysis (Fig. 6a). However, the removal of four different chemicals (chaetocin, oxamflatin, salermide, and delphinidin chloride) each inhibited the ability of pool #72 to improve *dmd* birefringence (Fig. 6a), indicating that these four compounds are involved in producing the *dmd* rescue effect of pool #72.

We next focused on the roles of chaetocin, oxamflatin, and salermide. We decided not to further test the role of delphinidin chloride primarily because it is not a component of the plate 2 row A compounds on which we decided to focus (delphinidin chloride is the plate 1 well C3 compound in pool #72) and also because it did not exhibit beneficial activity on its own (data not shown). To further test the roles of chaetocin, oxamflatin, and salermide in improving *dmd* muscle birefringence, we tested these three compounds together and in each pair-wise combination (Fig. 6b). The combination of oxamflatin and salermide significantly improved *dmd* mutant muscle birefringence, similarly to pool #72, whereas the other pair-wise combinations and the three-chemical combination did not (Fig. 6b). Figure 6c-f shows examples of the improved birefringence from animals treated with pool #72 (Fig. 6e) and the combination of oxamflatin+salermide (Fig. 6f). While these findings do not rule out possible contributions of delphinidin chloride and chaetocin, these results show that the combination of oxamflatin and salermide can mediate the beneficial effects of pool #72 in improving *dmd* mutant muscle birefringence.

### An independent laboratory test validates the beneficial effects of oxamflatin+salermide

To validate these effects of oxamflatin and salermide, we performed treatments from 1 dpf-4 dpf at an independent site, in the laboratory of Dr. Henry. We observed similar improvement of zebrafish *dmd* mutant muscle birefringence with oxamflatin+salermide treatments performed in the Henry Lab (Fig. 6g) as we observed with treatments performed in the Maves Lab (Fig. 6b). Dr. Henry’s lab also validated that TSA-improved zebrafish *dmd* mutant birefringence (Fig. 6h). By demonstrating reproducibility, these independent tests are a strong validation of the benefits of these small molecules, and in particular the novel oxamflatin+salermide combination, for *dmd* mutant zebrafish.

### Oxamflatin and salermide have dose-dependent effects, independent of dystrophin expression

**Fig. 7:**
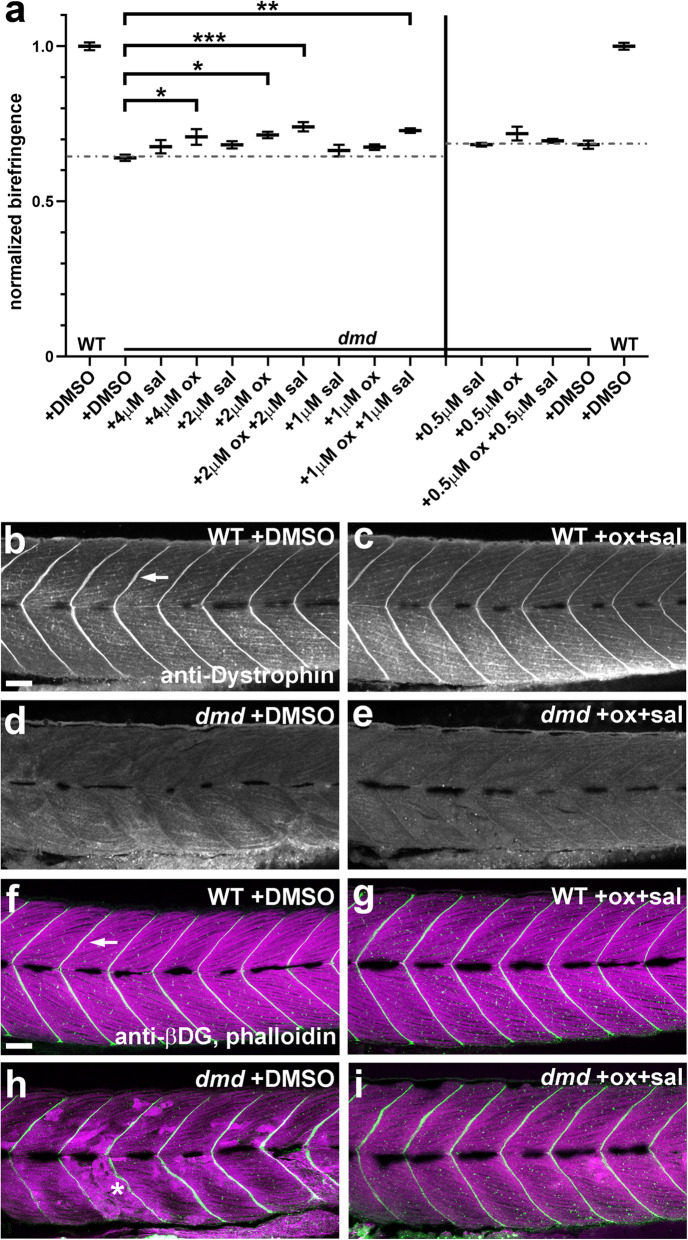
Oxamflatin and salermide have dose-dependent effects, independent of dystrophin expression. **a** Graph of average normalized pixel intensities for treatments of *dmd* mutants with doses of oxamflatin and salermide. Control treatment is 1% DMSO. Chemicals were used between 0.5 μM and 4 μM, over two separate experiments. For each treatment condition, *n* = 4 replicates, with 2-11 *dmd−/−* embryos in each replicate. Plot shows the average normalized pixel intensity for each of the 4 replicate pools for each treatment. The vertical line separates the treatment conditions from the two experiments, each of which has its own WT + DMSO and *dmd* + DMSO controls. The dashed lines represent the average normalized pixel intensity for all of the DMSO-treated *dmd* animals (*n* = 26 and *n* = 30). Error bars represent standard error. Significance was determined using a one-way ANOVA test comparing each treatment group to the *dmd* DMSO control group with Dunnett’s correction for multiple comparisons. *p ≤ 0.029, **p = 0.0029, ***p = 0.0007 compared to *dmd* DMSO control. **b-e** Confocal images of anti-dystrophin staining in the trunk musculature of 4 dpf **b** WT + DMSO, **c** WT + oxamflatin and salermide, **d** *dmd* + DMSO, and **e** *dmd* + oxamflatin and salermide larvae. Lateral views, anterior to the left. Arrow points to dystrophin expression in the vertical myoseptum. All *dmd+/+* animals showed normal dystrophin expression (WT + DMSO, *n* = 16; WT + ox+sal, *n* = 14) and all *dmd−/−* animals lacked detectable dystrophin expression (*dmd−/−* + DMSO, *n* = 23; *dmd−/−* + ox+sal, *n* = 18). Scale bar = 50 μm. **f-i** Confocal images of anti-β-dystroglycan (βDG) and phalloidin staining in the trunk musculature of 4 dpf **f** WT + DMSO, **g** WT + oxamflatin and salermide, **h** *dmd* + DMSO, **i** *dmd* + oxamflatin and salermide. Lateral views, anterior to the left. Arrow points to βDG expression (white) in the vertical myoseptum. Phalloidin staining of filamentous actin (magenta) shows the disrupted muscle structure in *dmd* mutants (* in **h**). All wild type animals (+/+ and +/−) showed normal β-dystroglycan expression (WT + DMSO, *n* = 27; WT + ox+sal, *n* = 26), and *dmd*−*/*− animals showed largely maintained β-dystroglycan expression (*dmd*−*/*− + DMSO, *n* = 9; *dmd*−*/*− + ox+sal, *n* = 14). Scale bar = 50 μm

To further address the effects of oxamflatin and salermide, we performed a dose-response analysis. We tested concentrations of oxamflatin and salermide, from 0.5 μM to 4 μM, both individually and in combination (Fig. 7a). As above, animals from *dmd+/−* crosses were treated from 1-4 days and then scored for muscle birefringence. We found that oxamflatin and salermide in combination at doses lower than 1 μM do not improve the *dmd* mutant birefringence phenotype, while a combination dose of 2 μM oxamflatin+salermide improves birefringence to a degree similar to that seen with the 1 μM combination dose (Fig. 7a). Four micromolar and 2 μM doses of oxamflatin alone improve the birefringence phenotype, but not as well as the 1 μM and 2 μM combination doses (Fig. 7a). Salermide alone did not improve muscle birefringence at any dose tested. We also found that a combination dose of 4 μM oxamflatin+salermide was toxic and caused lethality by 4 dpf (data not shown). These results reveal that, while oxamflatin alone can have beneficial effects, the combination of oxamflatin+salermide appears more effective at improving the *dmd* mutant birefringence phenotype.

We next asked whether oxamflatin and salermide are acting by upregulating dystrophin expression. Dystrophin expression is lost in *dmd*^*ta222a*^ mutants (Fig. 7d) [39]. One to four dpf treatments with 1 μM oxamflatin+salermide do not cause any noticeable upregulation of dystrophin expression (Fig. 7e). We also examined the expression of β-dystroglycan (βDG), a marker of the DAPC at the vertical myosepta in the zebrafish trunk [39, 56]. Previous studies have shown that βDG expression is largely still intact at the myosepta in *dmd* mutants (Fig. 7h) [39, 57], although see [58]. βDG-labeled myosepta, and overall muscle fiber structure, appear improved by 1 μM oxamflatin+salermide treatments in *dmd* mutants, and we do not observe any obvious upregulation of βDG or actin filaments (Fig. 7i). These results suggest that oxamflatin+salermide are acting independently of dystrophin upregulation and further demonstrate the improved muscle structure of *dmd* mutants caused by oxamflatin+salermide treatments.

### Oxamflatin and salermide together increase histone acetylation

**Fig. 8:**
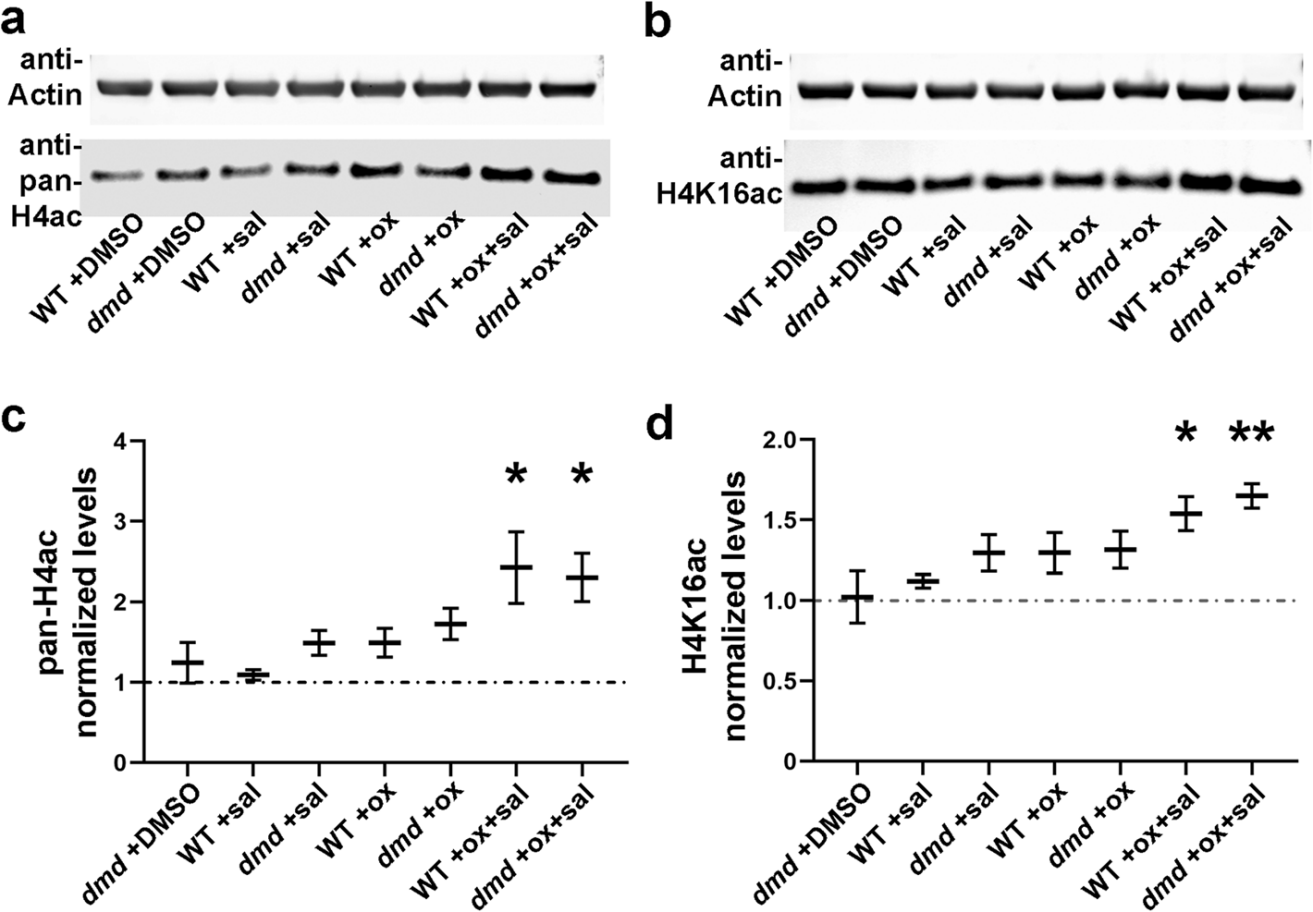
Oxamflatin and salermide together increase histone acetylation. **a-b** Representative Western blots of (**a**) histone H4 acetylated at lysines 5, 8, 12, and 16 (pan-H4ac) and (**b**) histone H4 acetylated specifically at lysine 16 (H4K16ac). Lysates are from genotyped 4 dpf larvae. **c-d** Quantification of Western blot analyses of levels of (**c**) pan-H4ac and (**d**) H4K16ac. Lysates were prepared from 4 replicate pools of animals for each treatment condition, with *n* = 4-10 animals per genotype per replicate. For each sample, pan-H4ac or H4K16ac signal was normalized to actin as a loading control. This value was then normalized to the WT + DMSO value within each set of samples. The plots show the average normalized signals of the 4 replicates. Error bars represent standard error. Significance was determined using a Student’s *t* test with Welch’s correction for unequal sample variances. In (**c**), *p < 0.05. In (**d**), *p = 0.015, **p = 0.003 compared to WT + DMSO control. Pan-H4ac levels are increased an average of 2.4× in oxamflatin+salermide-treated animals, and H4K16ac levels are increased an average of 1.6×

We next wanted to address the mechanisms by which oxamflatin and salermide might be working to improve the *dmd* mutant phenotype. Oxamflatin is an HDACi that inhibits class I and II HDACs [59, 60]. Salermide is a class III HDACi that inhibits the NAD + -dependent deacetylases SIRT1 and SIRT2 [61]. Oxamflatin and salermide have each been shown to cause increased histone acetylation in mammalian cell culture models [60, 61]. Salermide has also been shown to cause increased acetylation of lysine 16 of histone H4 (H4K16ac), a histone modification target of SIRT1 and SIRT2 [61–63]. We therefore asked whether oxamflatin and salermide treatments cause increased histone acetylation in *dmd* mutant zebrafish. We treated animals from *dmd+/−* crosses from 1-4 days and then collected animals at 4 dpf for protein lysates. Western analysis showed that treatments of 1 μM oxamflatin+salermide caused increased levels of pan-acetyl histone H4 and H4K16ac in both control and *dmd* mutant animals (Fig. 8a-d). However, 1 μM treatments of either oxamflatin or salermide did not increase levels of these histone marks (Fig. 8a-d). These results show that these small molecules are indeed acting as HDACi in zebrafish larvae and begin to reveal a possible mechanism for the activity of this chemical combination.

## Discussion

In this study, we performed a screen of epigenetic-modifying small molecules to identify compounds that prevented muscle degeneration in the zebrafish model of DMD. We developed a novel chemical-pooling approach to screen combinations of epigenetic small molecules, and we performed a pilot screen of 93 novel epigenetic small molecule pools. While our pilot screen identified several new candidate small molecule pools that have beneficial effects on zebrafish *dmd* mutants, our chemical-pooling strategy and subsequent analysis highlighted the activity of compounds from plate 2 row A (Fig. 4). We were able to resolve a novel small molecule combination, oxamflatin and salermide that significantly reduced the number of *dmd* animals with muscle lesions when compared to DMSO-treated controls. Our results provide support for further investigations of oxamflatin and salermide as potential therapeutic compounds for DMD. Our results also support additional screening of epigenetic small molecules for their beneficial effects in *dmd* mutant zebrafish.

Several previous large-scale chemical screens have demonstrated the potential of *dmd* mutant zebrafish for identifying new therapeutic compounds and targets [43, 45, 48, 49]. These studies, and our work here, took advantage of two key approaches, the birefringence assay and drug pooling, to efficiently screen large numbers of compounds. Assessing the percentage of “affected” animals in large-scale *dmd* mutant zebrafish chemical screens is efficient because the numbers of animals involved preclude performing genotyping. While there is potential for false negatives from these chemical screens, validation studies that incorporate animal genotyping and quantitative birefringence analysis, such as we use here with oxamflatin and salermide treatments, can alleviate concerns of false positives from chemical screens.

In contrast to previous screens that subdivided chemical libraries into unique chemical pools containing eight compounds each [45, 48], we wanted a strategy that would test each compound in combination with every other library compound. We designed a grid system in which each compound would be tested in combination with each of the other library compounds in at least 1 chemical pool (Fig. 1). By taking this novel drug pooling approach, we wanted to identify epigenetic small molecule combinations that are beneficial for *dmd* mutant zebrafish, and we identified the new combination of oxamflatin and salermide. While our particular drug pooling approach may not be easily feasible for larger library screens, taking similar systematic approaches to testing combinations of chemical library compounds could be fruitful for DMD and other diseases.

Based on results from our pilot screen, we focused our studies here on oxamflatin and salermide. Oxamflatin is an HDACi that inhibits class I and II HDACs and is chemically similar to TSA [59, 60]. Salermide is a class III HDACi that inhibits the NAD + -dependent deacetylases SIRT1 and SIRT2 [61], and so represents a new class of HDACi for DMD. In mammalian cell culture models, oxamflatin and salermide have each been shown to cause increased histone acetylation [60, 61]. Oxamflatin has been shown to inhibit cell proliferation and induce cell shape changes in cancer cell lines [60, 64, 65]. Salermide has been shown to inhibit cell proliferation and induce apoptosis in cancer cell lines, acting through reactivation of pro-apoptotic genes that are repressed by SIRT1-mediated H4K16ac deacetylation [61, 66, 67]. The H4K16ac mark is highly conserved, inhibits chromatin compaction, and has been associated with cell cycle progression, dosage compensation, cancer, and lifespan [68]. Our finding that oxamflatin+salermide caused increased levels of pan-acetyl histone H4 and H4K16ac in both wild-type and *dmd* mutant zebrafish (Fig. 8) shows that these HDACi compounds are having the expected biochemical effects on zebrafish larvae and also reveals that the combination of oxamflatin+salermide may be synergizing to activate specific histone marks.

In previous studies, overexpression of SIRT1 has been shown to ameliorate DMD pathophysiology in *mdx* mice [69], while salermide inhibition of SIRT1 has been shown to protect muscle cells against oculopharyngeal muscular dystrophy in *Caenorhabditis elegans* [70]. SIRT1 and SIRT2 can have opposing effects in promoting angiogenesis and in providing neuroprotective effects in neurodegenerative disease models [71, 72]. Future studies are needed to determine whether oxamflatin and salermide are working additively or synergistically to provide skeletal muscle benefits in zebrafish *dmd* mutants and whether they are acting through additional mechanisms that are independent of histone acetylation.

While many DMD small molecule therapies are being developed and entering clinical trials, some have difficulties showing efficacy in patients, possibly due to inadequate preclinical evaluation or targeting mechanisms too late in disease progression [7, 16, 19]. Underscoring the significance of this issue, a recent study was unable to replicate the benefits of the serotonin pathway modulator fluoxetine for *dmd* mutant zebrafish [73]. Here, we take advantage of the zebrafish model for DMD drug discovery and initial mechanistic validation. We also validated the effects of the epigenetic small molecules TSA and oxamflatin and salermide on *dmd* mutant zebrafish in an independent laboratory (Fig. 6). A critical next step will be to validate the effects of oxamflatin and salermide in mammalian DMD models, such as *mdx* mice, the *Dmd* rat, or human-induced pluripotent stem cell models of DMD [74–76]. We predict that small molecule validation in independent laboratories and in multiple model systems will increase the potential for future translation of epigenetic small molecule therapies and other pharmacological approaches for DMD.

The development of significantly beneficial therapies for DMD will likely involve the simultaneous use of a combination of therapies that target dystrophin and/or downstream pathological mechanisms [19, 24]. Epigenetic small molecules may represent a promising component of a DMD combination therapy. Epigenetic small molecules are outstanding candidates for a DMD pharmacological therapy for many reasons. Certain epigenetic small molecules have been shown to benefit DMD and other neuromuscular disorders [25, 77–80]. Several HDACi, including SAHA, and other epigenetic small molecules are already FDA-approved for use in cancers [81–83]. While some epigenetic small molecules have off-target or solubility issues, there is potential for optimizing dosing or using these chemicals as lead compounds for further analyses [81, 83]. In addition, there are growing numbers of examples showing that epigenetic small molecules synergize with each other or with other compounds to target different diseases, including cancer and heart disease [59, 81–85]. In our work here, we show an example of a combination of two epigenetic small molecules that are beneficial for *dmd* mutant zebrafish. Because of the advantages of the zebrafish animal model, we expect that *dmd* mutant zebrafish will be an increasingly important model for investigating combination therapies for DMD.

## Conclusions

- We developed a new chemical-pooling approach for screening combinations of small molecules in *dmd* mutant zebrafish.
- We identified a novel combination of epigenetic compounds, oxamflatin and salermide, that together improve skeletal muscle structural defects in *dmd* mutant zebrafish.
- We have taken an important step in small molecule validation for DMD by demonstrating that oxamflatin and salermide show beneficial effects on zebrafish *dmd* mutants in two independent laboratories.

## Data Availability

The datasets used and/or analyzed during the current study are available from the corresponding author on reasonable request.

## Abbreviations

βDG: β-dystroglycan
DAPC: Dystrophin-associated protein complex
DMD: Duchenne muscular dystrophy/dystrophin
DMSO: Dimethyl sulfoxide
dpf: Days post-fertilization
EM: Embryo medium
H4ac: Pan-acetylation of histone H4
H4K16ac: Acetylation of lysine 16 of histone H4
HDACi: Histone deacetylase inhibitors
hpf: Hours post-fertilization
PFA: Paraformaldehyde
TSA: Trichostatin A
WT: Wild-type
